# Chlorin E6-Curcumin-Mediated Photodynamic Therapy Promotes an Anti-Photoaging Effect in UVB-Irradiated Fibroblasts

**DOI:** 10.3390/ijms241713468

**Published:** 2023-08-30

**Authors:** Til Bahadur Thapa Magar, Shyam Kumar Mallik, Pallavi Gurung, Junmo Lim, Young-Tak Kim, Rajeev Shrestha, Yong-Wan Kim

**Affiliations:** Dongsung Cancer Center, Dongsung Biopharmaceutical, Daegu 41061, Republic of Korea

**Keywords:** photodynamic therapy, chlorin e6, curcumin, antioxidant, matrix metalloproteinase, photoaging

## Abstract

Skin photoaging due to ultraviolet B (UVB) exposure generates reactive oxygen species (ROS) that increase matrix metalloproteinase (MMP). Chlorin e6-photodynamic therapy (Ce6-PDT), in addition to being the first-line treatment for malignancies, has been shown to lessen skin photoaging, while curcumin is well known for reducing the deleterious effects of ROS. In the current study, PDT with three novel Ce6-curcumin derivatives, a combination of Ce6 and curcumin with various linkers, including propane-1,3-diamine for Ce6-propane-curcumin; hexane-1,6-diamine for Ce6-hexane-curcumin; and 3,3′-((oxybis(ethane-2,1-diyl))bis(oxy))bis(propan-1-amine) for Ce6-dipolyethylene glycol (diPEG)-curcumin, were studied for regulation of UVB-induced photoaging on human skin fibroblast (Hs68) and mouse embryonic fibroblast (BALB/c 3T3) cells. We assessed the antiphotoaging effects of Ce6-curcumin derivatives on cell viability, antioxidant activity, the mechanism of matrix metalloproteinase-1 and 2 (MMP-2) expression, and collagen synthesis in UVB-irradiated in vitro models. All three Ce6-curcumin derivatives were found to be non-phototoxic in the neutral red uptake phototoxicity test. We found that Ce6-hexane-curcumin-PDT and Ce6-propane-curcumin-associated PDT exhibited less cytotoxicity in Hs68 and BALB/c 3T3 fibroblast cell lines compared to Ce6-diPEG-curcumin-PDT. Ce6-diPEG-curcumin and Ce6-propane-curcumin-associated PDT showed superior antioxidant activity in Hs68 cell lines. Further, in UVB-irradiated in vitro models, the Ce6-diPEG-curcumin-PDT greatly attenuated the expression levels of MMP-1 and MMP-2 by blocking mitogen-activated protein kinases (MAPKs), activator protein 1 (AP-1), and tumor necrosis factor-α (NF-κB) signaling. Moreover, Ce6-diPEG-curcumin effectively inhibited inflammatory molecules, such as cyclooxygenase-2 (COX-2) and inducible nitric oxide synthase (iNOS) expression, while accelerating collagen synthesis. These results demonstrate that Ce6-diPEG-curcumin may be a potential therapy for treating skin photoaging.

## 1. Introduction

Skin aging is a natural process that occurs as we age. This is due to a combination of factors, including genetics, sun exposure, and lifestyle habits, such as smoking and a poor diet [1]. One of the main contributors to skin aging is exposure to ultraviolet (UV) radiation from the sun. UV radiation can damage the DNA in skin cells, leading to premature aging and an increased risk of skin cancer [2,3]. Normal skin aging can be characterized by fine wrinkles, reduced elasticity, and a thinning epidermis that appears smooth and pale as a result of the dermis’s progressive atrophy. It is believed that a decrease in the extracellular matrix (ECM) amount, particularly collagen in the dermis, is one of the main causes of dermal atrophy [4]. In aged skin, collagen production declines, and its degradation rises, resulting in an overall reduction in collagen content. Chronic UV-induced damage coupled with intrinsic aging results in photoaging and accounts for most age-associated changes in skin appearance [5]. UVB, whose wavelengths range from 280 to 320 nm, is detrimental to the skin as it causes sunburn, erythema, and photoaging, as well as malignant melanoma [6]. Photodamaged skin displays epidermal thickening, dermal elastosis, decreased collagen, increased matrix-degrading metalloproteinases, etc. [7].

The primary mechanisms underlying UVB-induced skin photoaging are the induction of oxidative stress, stimulation of an inflammatory response [8], abnormal expression of matrix metalloproteinases (MMPs), etc. [9]. Excessive UVB irradiation of skin cells produces a large amount of intracellular reactive oxygen species (ROS) that predisposes to an inflammatory cascade of skin photoaging, such as tumor necrosis factor-α (TNF-α)-induced inflammatory pathways through activation of nuclear factor-κB (NF-κB) [10]. In addition, ROS is known to initiate the activation of the mitogen-activated protein kinase (MAPK)-associated signaling pathway that is involved in the regulation of cell proliferation, differentiation, apoptosis, and inflammation. Therefore, targeting MAPK-regulated signaling pathways could potentially be an effective method for countering UVB-induced photoaging [11]. On the other hand, ROS is also vital to the regulation of collagen metabolism through the upregulation of MMPs, namely MMP-1, MMP-3, and MMP-9 in the human skin [12]. However, several previous reports showed the paradoxical functions of ROS as intracellular second messengers and signaling molecules in various intracellular processes [13].

Photodynamic therapy (PDT) is a medical treatment that involves the use of a photosensitizing agent and a specific wavelength of light to destroy target cells. PDT works by activating a photosensitizer (PS), a drug that is applied to the target tissue with a specific wavelength of light. This produces a form of oxygen (ROS) that is toxic to cells, leading to cell death [14,15]. Various treatment approaches have been designed to target and repair the molecular characteristics of aged skin [16]. Recently, clinical PDT has been employed for a variety of medical and cosmetic needs utilizing light-emitting diodes (LEDs) [17]. The advantage of LED light over lasers is that it is less invasive with relatively narrow spectral bandwidths, has high fluence rates, and has fewer side effects in targeted tissues [18]. PDT requires PS, oxygen, and light sources [19], while its adverse effects can be minimized by adjusting the dosage of the PS and the intensity of the light source [20]. Various PSs containing tetrapyrrole structures, such as chlorin e6 (Ce6), have an absorbance wavelength between 600 and 800 nm and exhibit no dark toxicity, along with rapid clearance from the body. Ce6-associated PDT has been known for its anti-bacterial, anti-inflammatory [21,22], anti-microbial, and anti-fungal [23], as well as anti-obesity [24] properties. Furthermore, curcumin is a widely studied nutraceutical belonging to the polyphenol family, acquired from the rhizome of the plant *Curcuma longa* (turmeric), a member of the ginger family [25]. Despite curcumin’s antioxidant, immunomodulatory, anti-inflammatory, anti-microbial, and anti-neoplastic properties [26], its anti-photoaging activity against UVA [27] and UVB-irradiation-induced alterations [28] has attracted us to this study. 

**Figure 1:**
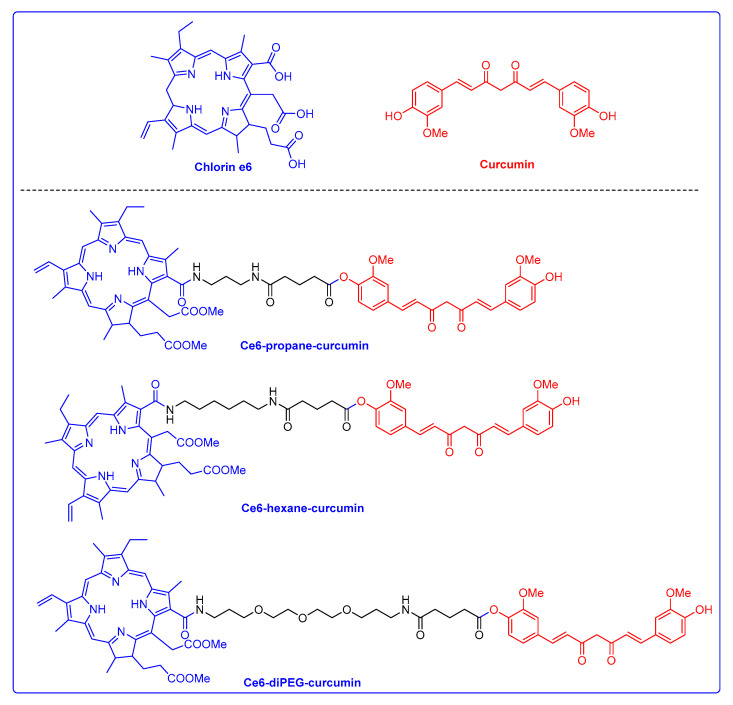
Structure of Chlorin e6, Curcumin, Ce6-propane-curcumin, Ce6-hexane-curcumin, and Ce6-diPEG-curcumin.

In our previous work, we have discussed the synthesis and characterization of Ce6-curcumin derivatives (Ce6-propane-curcumin, Ce6-hexane-curcumin, and Ce6-diPEG-curcumin) and their use in anticancer PDT studies (Figure 1) [29,30]. Herein, we have further examined the anti-photoaging efficacy and molecular mechanisms of Ce6-curcumin derivatives on UVB-irradiated in vitro models. 

## 2. Results

### 2.1. Photophysical Properties and Ex Vivo Distribution of Ce6-Curcumin Derivatives

**Figure 2:**
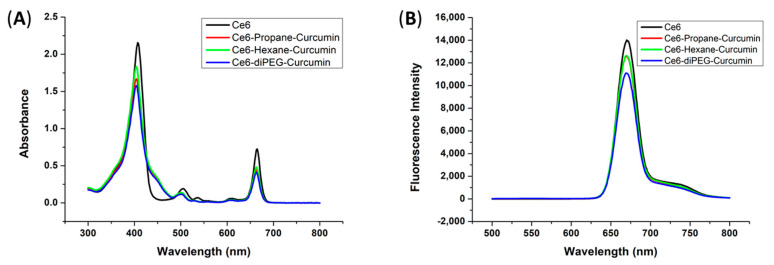
Photophysical properties of Ce6-curcumin derivatives: (**A**) absorption spectra of Ce6-curcumin derivatives; (**B**) fluorescence spectra of Ce6-curcumin derivatives.

The UV-vis absorption of compounds in 95% ethanol was analyzed and compared with the starting material Ce6, as shown in Figure 2A. The absorption spectra of all compounds were typical of Ce6 derivatives, with one Soret band and Q-bands. All the compounds Ce6, Ce6-hexane-curcumin, Ce6-propane-curcumin, and Ce6-diPEG-curcumin effectively absorbed the red light, with the major Soret peak at λ_max_ = 405–408 nm. The Soret band of Ce6 is sharp, while Ce6-curcumin derivatives have a broad peak due to curcumin conjugation. The conjugation of curcumin to Ce6 increases the absorption at a longer wavelength. Similarly, all the compounds show fluorescence at 650–700 nm (Figure 2B). 

**Figure 3:**
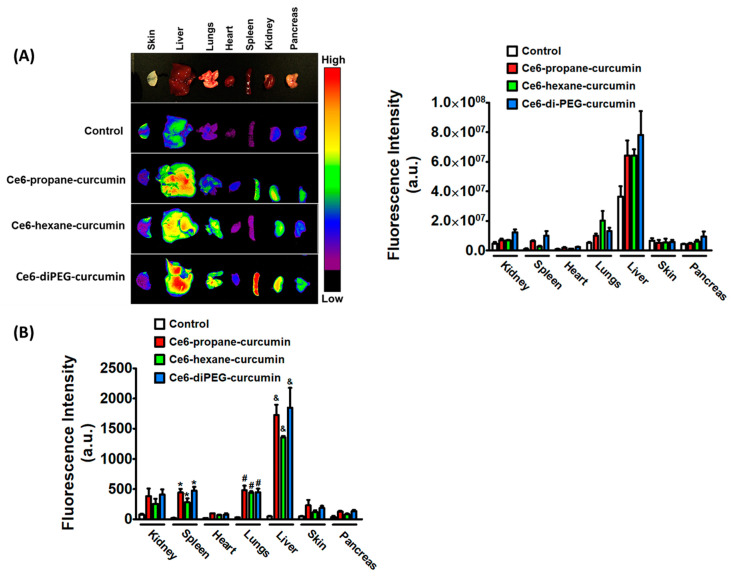
(**A**) Ex vivo fluorescence images of normal organs in ICR mice after 3 h post-injection of Ce6-propane-curcumin, Ce6-hexane-curcumin, and Ce6-diPEG-curcumin. (**B**) Biodistribution of the three Ce6-curcumin derivatives in the organ lysates after 3 h post-injection. The quantitation of fluorescence signals of Ce6 derivatives has been represented as a bar. Data were represented as mean ± SD from at least three independent experiments and were analyzed by one-way ANOVA with Tukey’s post hoc test: * *p* < 0.01, compared with vehicle-treated control of the spleen, ^#^ *p* < 0.01, compared with vehicle-treated control of the lungs, ^&^ *p* < 0.01, compared with vehicle-treated control of the liver.

To confirm the organ-specific signaling intensities of different Ce6-curcumin derivatives, we measured ex-vivo FLI in different organs of ICR mice after 3 h of intravenous drug administration. The drug interval time of 3 h before light irradiation in PDT for Ce6-curcumin derivatives can be explained by the pharmacokinetics and imaging study of Ce6 provided in Figures S1 and S2. Three hours after IV injection of 2.5 mg/kg of Ce6- hexane-curcumin, Ce6-propane-curcumin, and Ce6-diPEG-curcumin, ex-vivo images were obtained. The signal intensities from all Ce6-curcumin derivatives, as shown in Figure 3, confirmed that the liver showed a stronger signal compared to other organs. Among the derivatives, Ce6-diPEG-curcumin showed the highest fluorescence, followed by Ce6-propane-curcumin and Ce6-hexane-curcumin. Results of fluorescent measurements of Ce6-curcumin derivatives in different organs also demonstrated that Ce6-diPEG-curcumin could concentrate more strongly in various mouse tissues as compared to other Ce6 derivatives. Furthermore, the fluorescence intensity of Ce6-curcumin derivatives was highest in organs with a larger blood supply, including the liver and lungs.

### 2.2. Effect of Ce6-Curcumin Derivatives in the Viability of Hs68 and BALB/c 3T3 Cells 

**Figure 4:**
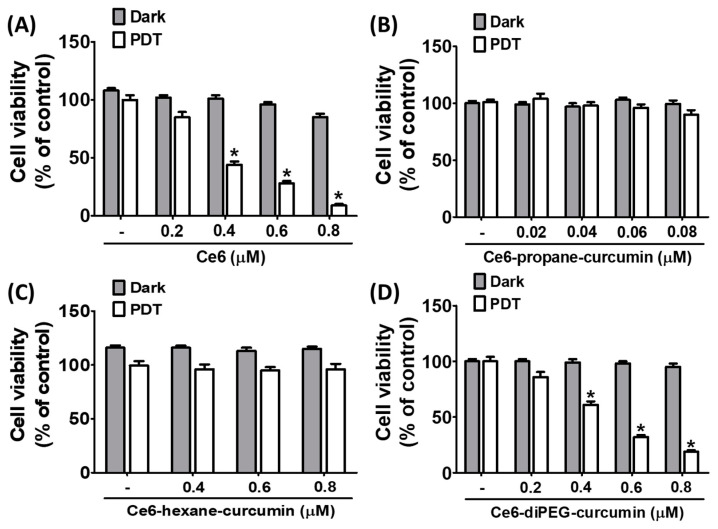
Effects of Ce6, Ce6-propane-curcumin, Ce6-hexane-curcumin, and Ce6-diPEG-curcumin with or without LED irradiation on cell viability. Hs68 cells were treated with Ce6 (200–1000 nM), Ce6-propane-curcumin (200–1000 nM), Ce6-hexane-curcumin (400–1000 nM), and Ce6-diPEG-curcumin (200–1000 nM) for 3 h, followed by irradiation of 20 J/cm^2^ or without irradiation for 24 h. Dark and phototoxicity of (**A**) Ce6, (**B**) Ce6-propane-curcumin, (**C**) Ce6-hexane-curcumin, and (**D**) Ce6-diPEG-curcumin against Hs68 cells, as detected by MTT assays. Data were represented as mean ± SD from at least three independent experiments and analyzed by one-way ANOVA with Tukey’s post hoc test, * *p* < 0.01, compared with vehicle-treated control.

To investigate the viability of Hs68 cells against various concentrations of Ce6 and Ce6-curcumin derivatives with or without LED light (20 J/cm^2^), the MTT assay was performed. The influences of Ce6 and Ce6-curcumin derivatives on the viability of Hs68 are shown in Figure 4 and Table 1. The results showed that the dark and phototoxicity of Ce6, Ce6-propane-curcumin, Ce6-hexane-curcumin, and Ce6-diPEG-curcumin (Figure 4A–D) displayed almost no cytotoxicity for Hs68 cells up to 1000 nM, whereas, with PDT, the phototoxicity of Ce6 and Ce6-diPEG-curcumin was increased to 615 and 692.6 nM, respectively (Figure 4A,D). However, it was confirmed that Ce6-propane-curcumin and Ce6-hexane-curcumin had no phototoxicity toward human dermal fibroblasts, Hs68, up to a dose of 1000 nM (Figure 4B,C). 

**Figure 5:**
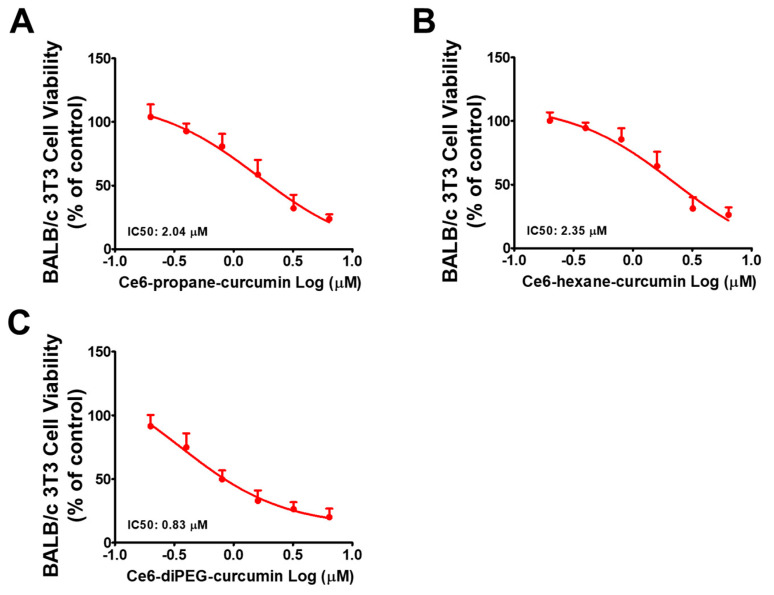
IC50 determination in BALB/c 3T3 cells. BALB/c 3T3 cells were treated with incremental concentrations of (**A**) Ce6-propane-curcumin, (**B**) Ce6-hexane-curcumin, and (**C**) Ce6-diPEG-curcumin derivatives for 3 h, followed by 660 nm laser irradiation of 20 J/cm^2^. MTT assay was used to evaluate viability after 24 h. IC50 values were calculated using GraphPad Prism 5 software.

Moreover, to clarify the cytotoxic effects of Ce6-curcumin derivatives with or without PDT against BALB/c 3T3 cells were assayed. Ce6-diPEG-curcumin showed cytotoxicity with an IC50 of 830 nM, while Ce6-propane-curcumin and Ce6-hexane-curcumin displayed minimal cytotoxicity potentials with IC50s of 2040 and 2350 nM at the tested concentrations (200 to 6400 nM), respectively (Figure 5A–C).

### 2.3. Effect of UVB on BALB/c 3T3 Cell Viability 

**Figure 6:**
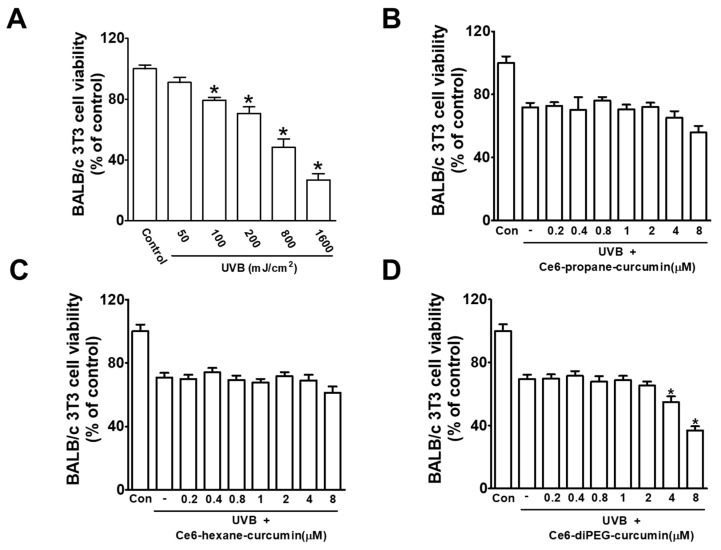
**The** effect of Ce6-curcumin derivatives on UVB-irradiated BALB/c 3T3 cells. (**A**) Cell viability of BALB/c 3T3 cells irradiated with different doses of UVB (50, 100, 200, 800, 1600 mJ/cm^2^) for 24 h. The effect of (**B**) Ce6-propane-curcumin, (**C**) Ce6-hexane-curcumin, and (**D**) Ce6-diPEG-curcumin in UVB-irradiated BALB/c 3T3 cells were determined by MTT assay. Data were represented as mean ± SD from at least three independent experiments and analyzed by one-way ANOVA with Tukey’s post hoc test, * *p* < 0.01, compared with UVB-treated groups.

To investigate the proper dose of UVB irradiation, BALB/c 3T3 cells were exposed to different doses of UVB (50, 100, 200, 800, 1600 mJ/cm^2^) for 24 h. UVB treatment decreased the cell viability in a dose-dependent manner (Figure 6A). Therefore, the dose of 200 mJ/cm^2^ was used in the subsequent in vitro studies to evaluate the protective effect of Ce6-PDT. The effect of Ce6-curcumin derivatives on BALB/c 3T3 cell viability was evaluated with the MTT assay. BALB/c 3T3 cells were irradiated with UVB (200 mJ/cm^2^) and incubated with Ce6-propane-curcumin, Ce6-hexane-curcumin and Ce6-diPEG-curcumin (0.2–8 μM) for 24 h, respectively. Cell viability was not affected by Ce6-propane-curcumin and Ce6-hexane-curcumin concentrations, while Ce6-diPEG-curcumin showed cytotoxicity from 4 μM concentrations (Figure 6B–D).

### 2.4. Antioxidant Potential of Ce6-Curcumin Derivativses

#### 2.4.1. ABTS Radical Scavenging Activity

The antioxidant activity of Ce6, Ce6-propane-curcumin, Ce6-hexane-curcumin, and Ce6-diPEG-curcumin was measured using ABTS assays with or without LED light (Table 2). The scavenging activity of Ce6 and its curcumin derivatives with or without LED treatments was assessed using the ABTS cation assay. The parent compound, Ce6 showed the lowest EC50 values (effective concentration of 50% inhibition of the radicals) of scavenging ABTS radicals with or without LED at about 13.58 ± 4.14 μM and 19.81 ± 1.57 μM, respectively, demonstrating the highest antioxidant activity, followed by Ce6-diPEG-curcumin (15.51 ± 0.01 μM and 22.33 ± 2.62 μM) > Ce6-propane-curcumin (18.90 ± 3.04 μM and 34.92 ± 8.16 μM) > Ce6-hexane-curcumin (22.36 ± 4.66 μM and 36.34 ± 4.02 μM). Ce6-diPEG-curcumin showed an EC50 value of almost 74% compared to the standard antioxidant, 5-ALA hydrochloride (60.28 ± 9.51 μM), demonstrating its excellent active oxygen scavenging ability and antioxidant activity. Curcumin’s antioxidative capacity was discovered to be superior to that of 5-ALA and Ce6-curcumin derivatives in dark conditions.

#### 2.4.2. ORAC (Oxygen Radical Absorbance Capacity) Assay

The antioxidant activity of Ce6, Ce6-propane-curcumin, Ce6-hexane-curcumin, and Ce6-diPEG-curcumin was measured using ORAC assays with or without LED light (Table 3). The result demonstrated that Ce6 and its derivatives antioxidant activity can be ranked in the following order at 1 μM concentration under dark conditions: Ce6-diPEG-curcumin (20.85 μM) > Ce6-propane-curcumin (20.11 μM) > Ce6 (18.5 μM) > Ce6-hexane-curcumin (15.97 μM). Similar to this, at 10 μM under dark conditions, Ce6 (47.72 μM) was followed by Ce6-curcumin-diPEG (30.32 μM), Ce6-propane-curcumin (26.83 μM), and Ce6-hexane-curcumin (20.19 μM) in terms of oxygen absorption capability. At both 1 μM and 10 μM, the reference control, 5-ALA, was undetectable. Ce6 also had the highest activity (22.11 μM) when exposed to LED light of 20 J/cm^2^ at 1 μM concentration, followed by Ce6-propane-curcumin (2.78 μM), Ce6-hexane-curcumin (2.26 μM), and Ce6-diPEG-curcumin (0.85 μM). The same order was obtained: Ce6 (53.85 μM) > Ce6-propane-curcumin (30.66 μM) > Ce6-diPEG-curcumin (27.98 μM) > Ce6-hexane-curcumin (24.17 μM) at 10 μM under LED light. As a result, we can draw the conclusion that Ce6 and Ce6-propane-curcumin demonstrated greater oxygen scavenging capacity under dark and LED conditions.

### 2.5. Gelatin Zymography

**Figure 7:**
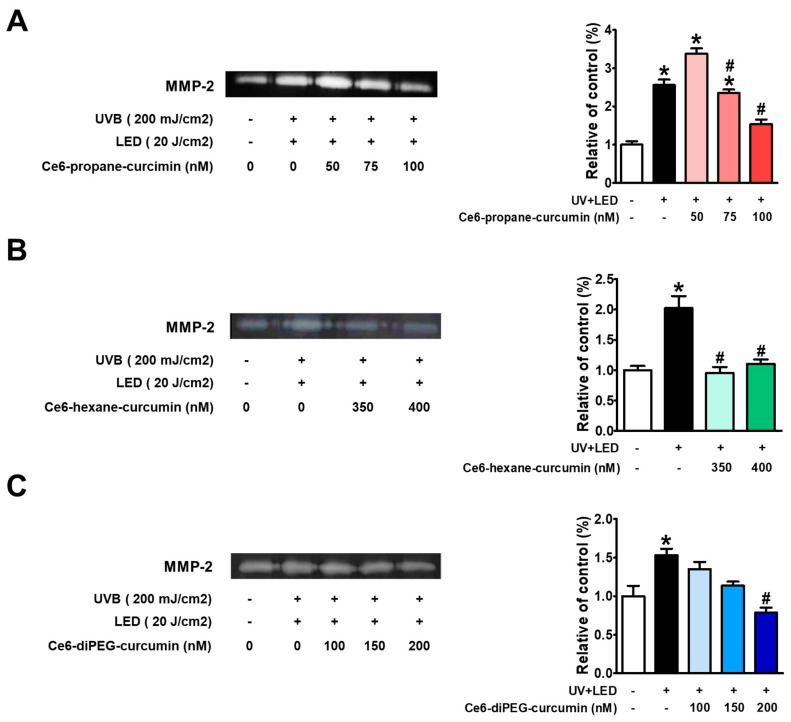
Effects of Ce6-propane-curcumin, Ce6-hexane-curcumin, and Ce6-diPEG-curcumin-mediated PDT on MMP-2 protein expression in UVB-exposed Hs68 cells. Hs68 cells were exposed to UVB-irradiation (200 mJ/cm^2^) and then treated with Ce6-propane-curcumin (50–100 nM), Ce6-hexane-curcumin (350–400 nM), and Ce6-diPEG-curcumin (100–200 nM) for 3 h, followed by irradiation of 20 J/cm^2^ or without irradiation for 24 h. Conditioned media were used for the measurement of MMP-2 protein levels by gelatin zymography for (**A**) Ce6-propane-curcumin, (**B**) Ce6-hexane-curcumin, and (**C**) Ce6-diPEG-curcumin. Data are expressed as mean ± SD from at least three independent experiments and were analyzed by one-way ANOVA with Tukey’s post hoc test, * *p* < 0.01 compared with control. ^#^ *p* < 0.05 compared with the UVB and LED-treated groups.

Since MMP-2 is a collagenase enzyme that plays an important role in photoaging, we examined the effect of Ce6-curcumin derivatives and LED treatment on the enzyme activities of UV-induced MMP-2 by gelatin zymography. On gelatin zymography, UVB irradiation led to the activation of MMP-2 in Hs68 cells, and this increase was inhibited by Ce6-curcumin derivatives along with LED treatments. On increasing the dose of the Ce6-curcumin derivatives, the MMP-2 was decreased (Figure 7A–C). Ce6-diPEG-curcumin at 200 nM among the other conjugates showed the highest attenuation of MMP-2 expression in UVB irradiated Hs68 human skin fibroblasts. While Ce6-propane-curcumin at a dose of 100 nM and Ce6-hexane-curcumin at 350 nM also showed significant reductions in UVB-induced MMP-2 expression. 

### 2.6. Collagen Assay

**Figure 8:**
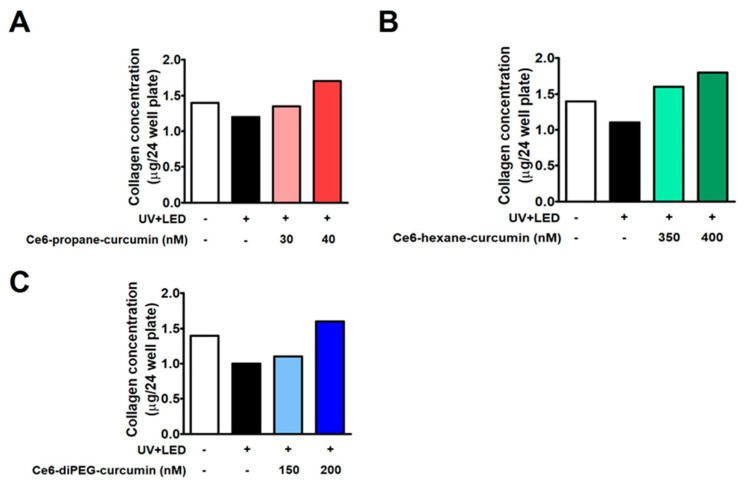
Effects of Ce6-propane-curcumin, Ce6-hexane-curcumin, and Ce6-diPEG-curcumin mediated PDT on collagen synthesis in UVB-irradiated Hs68 cells. Hs68 cells were exposed to UVB-irradiation (200 mJ/cm^2^) and then treated with Ce6-propane-curcumin (30–40 nM), Ce6-hexane-curcumin (350–400 nM), and Ce6-diPEG-curcumin (150–200 nM) for 3 h followed by irradiation of 20 J/cm^2^ or without irradiation for 24 h. Collagen synthesis was determined by using a collagen assay kit when Hs68 cells were treated with (**A**) Ce6-propane-curcumin, (**B**) Ce6-hexane-curcumin, and (**C**) Ce6-diPEG-curcumin-PDT by using a collagen assay kit. Each scale value is shown as the mean only.

Hs68 fibroblasts were treated with three Ce6-curcumin derivatives to promote cell collagen synthesis. The amount of collagen was measured through the Sircol collagen assay kit (Figure 8). The amount of collagen synthesis (1.3 μg) decreased in the UV-induced group, as seen in Figure 8A–C. However, when Ce6-curcumin derivatives were added with UVB irradiation of 200 mJ/cm^2^, the reduced collagen synthesis brought about by UVB was reversed in comparison to the control group. The experimental group irradiated with LED after treatment with Ce6-propane-curcumin at a concentration of 30–40 nM showed an increase in collagen synthesis in a dose-dependent manner in comparison to the control. Similar results were seen when Ce6-hexane-curcumin (350–400 nM) and Ce6-diPEG-curcumin (150–200 nM) were irradiated with LED.

### 2.7. Effects of Ce6-Curcumin-Derivatives-PDT on MAPK, NF-κB and AP-1 Signaling in 3T3 Cells

**Figure 9:**
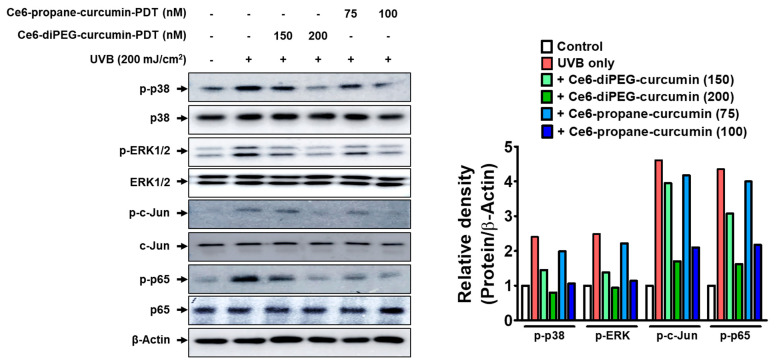
Effect of Ce6-curcumin derivatives-PDT on MAPK, NF-κB and c-JUN signaling proteins. Protein expressions of p-p38, p-38, p-ERK, ERK, p-c-JUN, c-JUN, p-p65 and p65 in the UVB-induced murine 3T3 fibroblast cell line after Ce6-diPEG-curcumin (150 and 200 μΜ) and Ce6-propane-curcumin (75 and 100 μΜ)-PDT treatment. Representative bars are expressed as mean only.

One of the major factors of UVB-induced skin aging is inflammation-mediated skin aging [31]. In UVB-induced skin photoaging, increased intracellular ROS can promote MMP production by activating the MAPK, AP-1, and NF-κB pathways. Hence, the phosphorylation levels of MAPK, NF-κB, and AP-1 subunits were measured after Ce6-diPEG-curcumin-PDT and Ce6-propane-curcumin-PDT treatment in the background of UVB treatment in order to explore the mechanism of Ce6-curcumin derivatives antiwrinkle effects. Ce6-diPEG-curcumin-PDT and Ce6-propane-curcumin-PDT lowered the phosphorylation levels of ERK and p38 MAPKs but not those of JNK MAPK. UVB irradiation resulted in increases in p-NF-κB p65 and p-c-JUN, but treatment with Ce6-diPEG-curcumin (150 and 200 μM) and Ce6-propane-curcumin (75 and 100 μM) followed by laser irradiation greatly reversed this trend compared to the control (Figure 9). These results suggest that PDT-associated with Ce6-diPEG-curcumin and Ce6-propane-curcumin can inhibit the UVB-induced activation of the ERK/p38 MAPK pathway and decrease MMP expression, thereby increasing collagen synthesis.

### 2.8. Effects of Ce6-Curcumin-Derivatives-PDT on the Expressions of NF-κB-Dependent Genes

**Figure 10:**
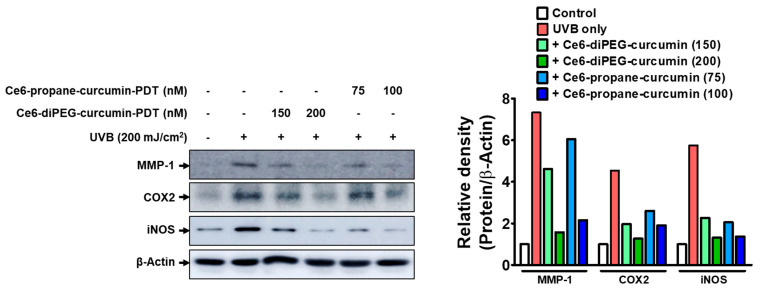
Effect of Ce6-curcumin derivatives-PDT on UVB-induced COX-2 and iNOS expressions. COX-2 and iNOS protein expressions were detected using western blotting in a UVB-induced murine 3T3 fibroblast cell line after Ce6-diPEG-curcumin (150 and 200 μΜ) and Ce6-propane-curcumin (75 and 100 μΜ)-PDT treatment. Representative bars are expressed as mean only.

We further investigated the expressions of COX-2, TNF-α, and MMP-1 to ascertain the effect of UVB on the expressions of NF-κB and AP-1-dependent genes. These genes have an NF-κB and AP-1 functional binding site in their promoter regions and are linked to inflammation. As seen in Figure 9, UVB upregulated the COX-2 and TNF-α protein levels, while pretreatments with Ce6-diPEG-curcumin (150 and 200 μΜ) and Ce6-propane-curcumin (75 and 100 μΜ) downregulated these increases in fibroblast cells. The Ce6-diPEG-curcumin decreased the expression levels of iNOS and COX-2 to below those seen in the normal control at a concentration of 200 nM (Figure 10). These results confirm that Ce6-diPEG-curcumin and Ce6-propane-curcumin can modulate NF-κB and AP-1 activation and the expression of their genes.

### 2.9. Molecular Docking Analysis

Collagenase is an enzyme that breaks down collagen, a protein that is a major component of the skin responsible for elasticity and strength. As we age, collagen production decreases, and collagenase activity increases, leading to the breakdown of these tissues. This process contributes to the appearance of wrinkles, sagging skin, and other signs of aging. Additionally, collagenase also plays a role in the development of age-related diseases, such as osteoarthritis. Collagenase inhibitors are substances that can block the activity of collagenase enzymes, thereby preventing the breakdown of collagen in the skin. Therefore, we have decided to analyze the binding affinity of Ce6-curcumin derivatives to the collagenase enzyme using a web-based server for protein-ligand docking program (CB-Dock2) [32]. 

**Figure 11:**
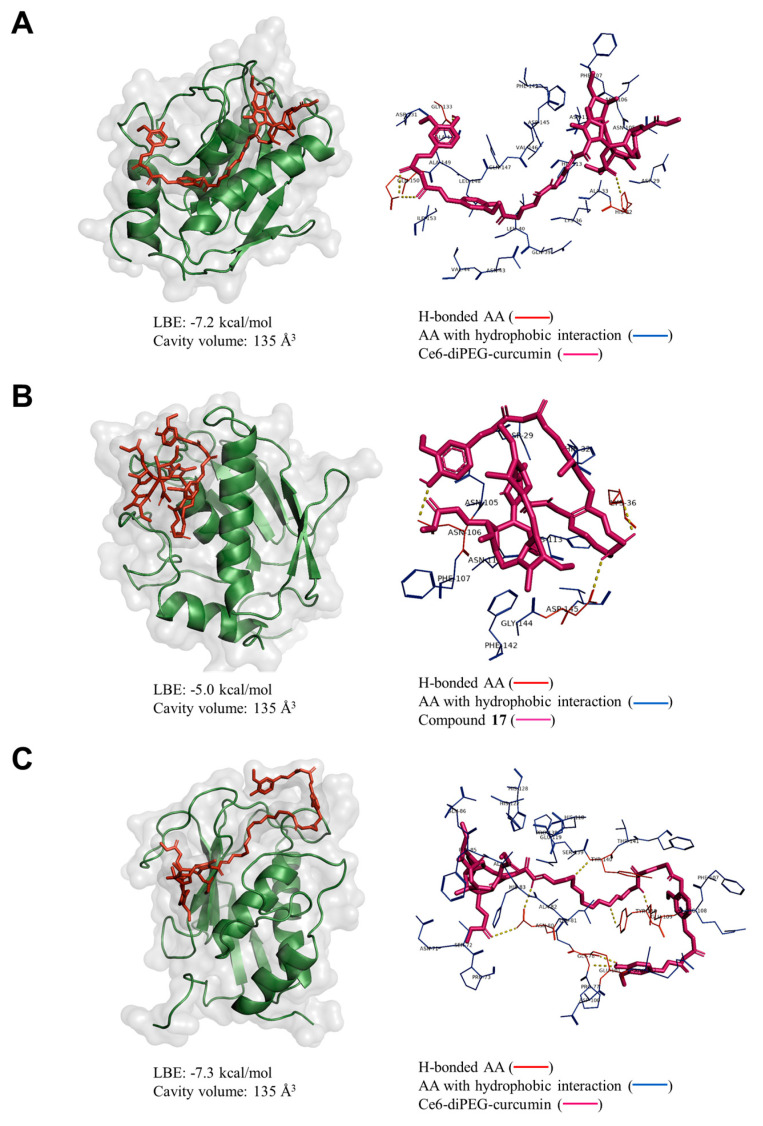
Binding affinity of (**A**) Ce6-propane-curcumin, (**B**) Ce6-hexane-curcumin, and (**C**) Ce6-diPEG-curcumin derivatives to the collagenase (PDB code: 2TCL). The lowest binding energy (LBE) and pocket volume were specified. Amino acids (AA) with polar and non-polar interactions were marked red and blue color, respectively (hydrogen bonds were represented as yellow dotted lines).

Among them, the Ce6-diPEG-curcumin conjugate was bound to the protein with the lowest binding energy (LBE) of −7.3 kcal/mol (Cavity volume: 135 Å). It was bound to the GLU 101, GLU 109, GLY 78, ASN 80, TYR 110, and TYR 140 residues of the enzyme via hydrogen bonding. Similarly, Ce6-propane and –hexane derivatives were bound to the same pocket of collagenase with LBE of −7.2 and −5.0 kcal/mol, respectively (Figure 11A–C). The binding pose of Ce6-curcumin derivatives to the MMP-2 displayed a similar result (Figure S4). This result supports the better anti-photoaging efficacy of Ce6-propane and –diPEG derivatives, as indicated by the above results.

## 3. Discussion

Photoaging is the major effect of extrinsic skin aging caused by exposure to UV light. UVB affects the epidermis and the upper layer of the dermis [33]. Photoaged skin is identified by degradation of the dermal ECM, reduced collagen synthesis, enhanced collagen degradation, loss of hyaluronic acid (HA), and disorganized elastin [34]. Previous studies have shown that PDT has proven useful for the treatment of sun-damaged skin. 5-Aminolevulinic acid (ALA)-PDT is known to improve fine wrinkles and tighten slackness in photoaged skin through the upregulation of collagen production [35]. PDT has been shown to cause apoptosis, necrosis, and exfoliation in highly proliferated epidermal cells. Besides, it can also lead to a local immune response, initiating repair mechanisms and promoting new collagen production due to increased procollagen I and III [36].

In addition, several research groups have reported on the PDT-enhancing activities of antioxidant molecules, such as ascorbate, 3(2)-tetra-butyl-4-hydroxyanisole, epigallocatechin-3-gallate, α-tocopherol, and trolox ([±]-6-hydroxyl-2,5,7,8-tetramethyl chromane-2-carboxylic acid, a water-soluble vitamin E analog [37]. Curcumin, widely known for its antioxidant, anti-inflammatory, and antitumor effects, is safe for humans at therapeutic doses in Phase I trials [38] and aids in collagen deposition, improves wound healing, and regulates skin disorders [24]. Furthermore, curcumin is known to block the production of UVB-induced ROS in fibroblasts, hence inhibiting the expression of MMP-1/3 [39]. Furthermore, curcumin encapsulated in elastic vesicles was found to have strong antioxidant activity and high radical scavenging capacity that inhibit UV-induced skin photoaging in mice [40]. Accordingly, we designed Ce6-curcumin conjugate (Ce6-monoPEG-curcumin) and discussed how it shields hairless mice and UVB-irradiated fibroblasts from photoaging [41]. Based on these intriguing results, we designed the new Ce6-curcumin derivatives by conjugating Ce6 and curcumin with different linkers (propane-1,3-diamine, hexane-1,6-diamine, and 3,3’-((oxybis(ethane-2,1-diyl))bis(oxy))bis(propan-1-amine) to obtain Ce6-propane-curcumin, Ce6-hexane-curcumin, and Ce6-diPEG-curcumin [29]. We also investigated how they work in conjunction with PDT to prevent photoaging in Hs68 cells.

In the present study, we determined the cytotoxic effects of Ce6-curcumin derivative-associated PDT against human Hs68 dermal fibroblasts by MTT assay. We selected this cell line because, according to Singh et al., 2022, when exposed to UV light, they express increased levels of matrix-degrading MMPs [42], thus making them good photoaging models. All three Ce6-curcumin derivatives exhibited low dark toxicity up to 1000 nM, much like their parent molecule, Ce6. Ce6-diPEG-curcumin-mediated PDT demonstrated phototoxicity at 692.6 nM, whose trend is similar to Ce6-PDT (615 nM) (Table 2). PDT with Ce6-propane-curcumin and Ce6-hexane-curcumin was not toxic at tested concentrations up to 1000 nM. Hence, Ce6-curcumin derivatives had no phototoxicity against Hs68 cells. Among the tested conjugates, Ce6-diPEG-curcumin-mediated PDT showed preferential cytotoxicity against BALB/c 3T3 cells with IC50 values of 0.8 μM, while Ce6-propane-curcumin-mediated PDT and Ce6-hexane-curcumin-mediated PDT had IC50 values of 2.04 and 2.35 μM, respectively. Ce6-propane-curcumin and Ce6-hexane-curcumin did not exhibit any toxicity, while Ce6-diPEG-curcumin showed minimal toxicity with tested concentrations against UVB-irradiated BALB/c 3T3 cells. In addition, we have also determined whether Ce6-curcumin-conjugate was phototoxic or not by the 3T3 NRU cytotoxicity assay (Figure S3 and Table S1).

In addition, we also determined its anti-oxidative activity in the presence of PDT using the ABTS and ORAC assays. Generally, oxidative damage induced by free radicals accelerates the aging process [43]. Since antioxidant activity is closely related to anti-aging activity, as mentioned above, all three Ce6-curcumin derivatives performed better in vitro antioxidant activity than 5-ALA, a standard antioxidant. However, Ce6-diPEG-curcumin alone demonstrated superior radical scavenging activity among them, which was lessened when used with PDT. Oxygen scavenging capacity (ORAC assay) assesses antioxidant inhibition of peroxyl radical-induced oxidations and reveals radical chain-breaking antioxidant activity through H-atom transfer [44]. In this assay, Ce6-curcumin derivatives showed a slightly different pattern, with Ce6-propane-curcumin demonstrating a higher oxygen scavenging capacity than Ce6-hexane-curcumin and Ce6-diPEG-curcumin at both 1 and 10 nM concentrations. This result is consistent with our previous work that was conducted on hairless mice and fibroblasts exposed to UVB radiation to study the antioxidant effect of Ce6-monoPEG-curcumin-mediated PDT [41].

To elucidate the molecular mechanisms by which Ce6-curcumin derivatives protect UVB-induced Hs68, we have analyzed the MMP-2 gelatin activity by zymography. As evident, high UVB-irradiation-induced ROS mediates different pro-inflammatory factors, which play a key role in the inflammatory signaling pathway during photoaging [45]. During inflammation-associated aging, ROS plays a crucial role in collagen metabolism, which results in the activation of MAPK and AP-1 signaling pathways and further regulates the expression of MMP-1, MMP-2, and MMP-9 [46,47]. Since MMPs are a relevant driver in photoaging, therapies aimed at reducing MMPs can potentially counteract photoaging, and topical MMP inhibitors have been proven to be helpful in inhibiting UVB-induced wrinkles [48]. In this study, we found that MMP-1 and MMP-2 were upregulated by UVB irradiation in fibroblast cells but decreased when these cells were treated with Ce6-curcumin derivative-PDT. Among the tested derivatives, Ce6-diPEG-curcumin has shown better results in reducing MMP-1 and MMP-2. We also obtained a concentration-dependent reduction of UVB-induced MMPs, as shown in our previous study with Ce6-PEG-curcumin-mediated PDT [41]. Further, in order to elucidate the molecular mechanism of Ce6-curcumin-derivatives-PDT for lowering MMPs, we examined the UVB-induced phosphorylation of MAPK, NF-κB, and subunits of AP-1. In our study, Ce6-diPEG-curcumin and Ce6-propane-curcumin-PDT decreased the UVB-stimulated phosphorylation of c-Jun, as well as of p65NF-κB. In addition, treatment with Ce6-diPEG-curcumin and Ce6-propane-curcumin-PDT suppressed the UVB-induced phosphorylation of p38 and ERK but not JNK. Thus, our results suggest that Ce6-curcumin derivatives-PDT prevent photoaging by inhibiting the p38 and ERK MAPK signaling pathways and thereby reducing c-Jun, p65NF-κB, MMP-1, and MMP-2 expression. In prior studies, we have found that UVB exposure to the skin induces components of AP-1 and NF-κB [49,50]. Therefore, in the present study, we found that Ce6-diPEG-curcumin and Ce6-propane-curcumin-PDT augmented the expression of COX-2 and iNOS and subsequently suppressed inflammatory signals.

Collagen, as an extracellular matrix, has a special role in wrinkle formation, as well as wound healing. Therefore, changes associated with collagen are an important hallmark of UV irradiation-induced aging. In our study, collagen production that had been reduced by UVB was recovered by Ce6-curcumin derivative-mediated PDT as compared to the control. PDT with all three Ce6-curcumin derivatives exhibits enhanced collagen production against UVB-induced changes in human dermal fibroblasts. However, Ce6-propane-curcumin-mediated PDT showed better collagen synthesizing activity than with the other two derivatives. Ce6-curcumin derivatives with PDT might have enhanced photorejuvenation through the generation of ROS, resulting in prolonged activation of the extracellular signal-regulated kinase (ERK) pathway [51] and further increased collagen expression. In addition, it has been known that PDT results in apoptosis or necrosis of exfoliated dermal cells and generates a local immune response, thus promoting repair mechanisms, fibroblast proliferation, and collagen synthesis [13]. Our findings are in line with those obtained using PDT in conjunction with electrodynamic treatment on SKH-1 hairless mice, which showed enhanced collagen I expression and decreased MMP-2 expression [52]. In addition, molecular docking simulations rendered that curcumin derivatives and collagenase were found to have good binding affinities in the order of Ce6-diPEG-curcumin > Ce6-propane-curcumin > Ce6-hexane-curmin conjugates, respectively. Based on the current docking result of curcumin and its derivatives, along with earlier findings [53,54,55], we can suggest that Ce6-curcumin-conjugates have the potential to inhibit collagenases. Altogether, our results demonstrate that Ce6-curcumin derivative-PDT protected against UVB-induced photoaging-associated damages in Hs68 cells through strong antioxidant action reduced MMP-2 expression, and promoted collagen synthesis.

## 4. Materials and Methods

### 4.1. Synthetic Chemistry

The Ce6-curcumin derivatives were synthesized by utilizing N-boc-monoprotected propane, hexane, and 4,9-dioxa-1,12-dodecanediamine (diPEG) linkers via a previously reported method [29]. Dimethyl ester of Ce6 was obtained by selective esterification of Ce6 in the presence of 5% H_2_SO_4_ in methanol. The mono-carboxylic acid derivative of curcumin was synthesized by reacting curcumin with glutaric anhydride in the presence of 4-dimethylaminopyridine (DMAP) and triethylamine (TEA). The dimethyl ester of Ce6 was activated using 1-hydroxybenzotriazole (HOBt) and 1-ethyl-3-(3-(dimethylamino)propyl)-carbodiimide hydrochloride (EDCI), then reacted individually with N-boc-monoprotected propane, hexane, and diPEG, followed by deprotection with trifluoroacetic acid to obtain Ce6-propane, Ce6-hexane, and Ce6-diPEG intermediates. Finally, curcumin monocarboxylic acid was coupled with Ce6-propane, Ce6-hexane, and Ce6-diPEG intermediates in the presence of HOBt and EDCI to obtain Ce6-propane-curcumin, Ce6-hexane-curcumin, and Ce6-diPEG-curcumin derivatives. Silica gel column chromatography was used for the purification of synthesized derivatives.

### 4.2. Photophysical Properties and Comparative Biodistribution Study of Ce6-Curcumin Derivatives

UV-vis spectra of Ce6 and Ce6-curcumin derivatives were analyzed in 95% ethanol (Duksan, Ansan-si, South Korea) at a concentration of 10 ppm (Thermo-scientific, Skanlt software 5.0, Waltham, MA, USA). The data were corrected for solvent background by the instrument’s calibration using 95% ethanol as a blank. The absorption spectra of the samples were obtained using a cuvette having a 1 cm path length in the range of 300–800 nm at a 1 nm interval. The fluorescence intensities of Ce6 and Ce6-curcumin derivatives were recorded on a Spark^®^ multimode microplate reader (Tecan Trading AG, Männedorf, Switzerland) at the emission wavelength range of 500–800 nm. The samples for fluorescence analysis were prepared in 95% ethanol at a concentration of 10 ppm.

### 4.3. Cell Culture

Hs68 human dermal fibroblasts were purchased from the American Type Culture Collection (Manassas, VA, USA), while BALB/c 3T3 cell lines were obtained from the Korean Cell Line Bank (KCLB, Seoul, South Korea). These cells were cultured in monolayers at 37 °C in a 5% CO_2_ incubator in Dulbecco’s modified Eagle’s medium (DMEM) containing 10% fetal bovine serum.

### 4.4. Cell Viability Assay (MTT Assay)

Hs68 and BALB/c 3T3 cell viability was measured using a 3-[4,5-dimethylthylthiazol-2-yl]-2,5 diphenyl tetrazolium bromide (MTT) assay. The cells were cultured in 24-well plates at a density of 3 × 10^4^ cells per well or BALB/c 3T3 cells at a density of 1 × 10^4^ cells per well. The cells were then treated with various concentrations of Ce6-curcumin derivatives (Ce6-propane-curcumin, Ce6-hexane-curcumin, and Ce6-diPEG-curcumin) for 30 min in the dark. The Hs68 cells were then exposed to LED light (660 nm; 89 W/m^2^; 20 J/cm^2^) for 37 min 27 s and incubated for 3 h. The Ce6-curcumin derivatives containing DMEM medium were then changed with serum-free DMEM medium, and the Hs68 cells were incubated for 24 h 30 min. The cells were washed and treated with MTT, after which the plates were incubated at 37 °C in the dark for 2 h. After the formation of formazan, 100 µL of DMSO was added, and the absorbance was measured at 570 nm using a microtiter plate reader (Thermo-scientific, Skanlt software 5.0, Waltham, MA, USA)

### 4.5. Cell Viability and UVB

BALB/c 3T3 cells were seeded at a density of 1 × 10^4^ cells/well in 96-well plates for 24 h. The culture medium was replaced with phosphate-buffered saline (PBS), and the cells were pretreated with UVB (200 mJ/cm^2^) before being incubated for 24 h incubation in an FBS-free medium containing different concentrations of Ce6-curcumin derivatives. Cell viability was measured by MTT assay.

### 4.6. Antioxidant Activity 

#### 4.6.1. ABTS Radical Scavenging Activity

The ABTS radical scavenging activity of Ce6-curcumin derivatives was measured by modifying the method of Van den Berg et al. [56]. Samples were dissolved in 2.5 mM potassium persulfate for more than 16 h to develop 7 mM 2,2′-azinobis-(3-ethylbenzothiazoline-6-sulfonic acid diammonium salt) (ABTS), and ethanol was diluted to obtain an absorbance of 0.3 at 620 nm. Each composite sample was diluted by concentration, and 100 μL of the sample was put into each well along with 50 μL each of ABTS reagent and solvent; reacted at room temperature for 10 min, and absorbance was measured at 620 nm.

ABTS activity inhibition (%) = 100 − [test group-blank]/(control group-blank) × 100]

#### 4.6.2. ORAC (Oxygen Radical Absorbance Capacity) Assay

The oxygen radical absorbance capacity value of Ce6-curcumin derivatives (1 µM and 10 µM) was measured by modifying the experimental method of Ou et al. [57]. The ORAC assay is based on the scavenging of peroxyl radicals generated by 2,2′-azobis(2-methylpropioamidine) dihydrochloride (AAPH), which prevents the degradation of the fluorescein probe. The ORAC values were calculated as the area under the curve (AUC) and expressed as µM of Trolox equivalent (TE).

### 4.7. Gelatin Zymography

MMP-2 activity in cell culture medium was evaluated by gelatin zymography assay as described [58]. After UVB (200 mJ/cm^2^) irradiation, Hs68 cells were cultured for 24 h. Ce6-curcumin derivatives were diluted into a medium containing 1% FBS and cultured for 30 min. After 30 min, the LED was irradiated with 20 J/cm^2^, cultured for 3 h, washed with 1× PBS, and 300 μL of FBS-free medium was added and cultured for 44 h. SDS-PAGE (Sodium dodecyl sulfate-polyacrylamide gel electrophoresis) was performed. After electrophoresis, the gel was washed with tertiary distilled water, soaked in 50 mL of 2.5% Triton X-100 solution, washed twice for 30 min each, and then washed with 50 mL of reaction buffer (50 mM Tris-HCl, 10 mM CaCl_2_, 50 mM NaCl, 0.05% Brij 35, pH 7.6) at 37 °C overnight. The activity of MMPs was measured by quantifying the transparent band by proteolytic activity after staining with Coomassie Brilliant Blue staining solution and further distaining with water, methanol, and acetic acid (50:40:10).

### 4.8. Collagen Assay

In order to measure the effect of Ce6-curcumin derivatives on the promotion of collagen synthesis in Hs68 cells by using the Sircol collagen assay (Biocolor, Belfast, UK) kit. UVB-exposed Hs68 cells were incubated for 44 h in the dark after Ce6-curcumin derivative-mediated PDT. Following this, 1 mL Sirius red dye, an anionic dye that reacts specifically with the basic side chain groups of collagens under the assay conditions, was added to 300 µL of cell culture medium supernatant and incubated with gentle rotation for 30 min at room temperature. After centrifugation, the pellet was washed with ice-cold acid-salt wash reagent released in alkali reagent, and the absorbance at 570 nm was measured using an ELISA reader (Sunrise, Tecan, Männedorf, Switzerland). The amount of collagen was calculated based on a standard curve obtained with the standard bovine type I collagen supplied with the kit.

### 4.9. Western Blot

The cells were lysed with RIPA lysis buffer containing protease inhibitor cocktails (Gen-DEPOT, Katy, TX, USA). Cell lysates were vortexed and centrifuged at 13,000 rpm for 15 min at 4 °C. Protein concentration was determined using the Bradford protein assay kit, and the same amount of protein was separated by SDS-PAGE gel. Then, proteins were transferred to a polyvinylidene fluoride (PVDF) membrane. Further, the membranes were blocked with a blocking solution containing 5% skim milk in Tris-buffered saline containing Tween 20 (TBST) for 1 h and incubated overnight with the primary antibody diluted in 5% skim milk in TBST at 4 °C. Then, the membrane was rinsed with TBST for 30 min and incubated with horseradish peroxidase-conjugated secondary antibodies in a blocking solution for 1 h. All bands were visualized by using an enhanced chemiluminescence (ECL) kit (Pierce, Rockford, IL, USA) under a luminescent image analyzer (Amersham, GE Healthcare, Chicago, IL, USA). The primary antibodies against MMP-1 were obtained from Thermo Fischer Scientific Inc. (NYSE: TMO). Antibodies directed against p-p65, p65, p-p38, p38, p-ERK, ERK, p-c-JUN, and c-JUN were purchased from Cell Signaling Technology Inc. (Beverley, MA, USA). COX-2 and β-Actin antibodies were from Santa Cruz Biotechnology (Santa Cruz, CA, USA), while TNF-α antibodies were procured from Novus Biologicals, Inc. (Centennial, CO, USA).

### 4.10. Animal Care

For the biodistribution experiment, ICR mice (n = 3) at the age of six weeks were procured from Orient Bio (Sungnam, South Korea) and kept in a pathogen-free environment at 28 °C in the controlled animal house facility of Dongsung Cancer Center, Daegu. They were subjected to a seven-day acclimation period with a 12-h alternate light and dark cycle. Each experimental group consisted of randomly selected mice with the same weights. All the mouse experiments were conducted following approval from the Institutional Animal Care and Use Committee of the Dongsung Cancer Center under protocol IACUC #ds002106117-2 [30]. 

### 4.11. Ex Vivo Imaging of Ce6 and Ce6-Curcumin Conjugates in Mice

Biodistribution analysis for Ce6 and Ce6-curcumin derivatives was calculated by measuring tissue fluorescence. Animals were injected intravenously either with or without Ce6, Ce6-propane-curcumin, Ce6-hexane-curcumin, and Ce6-diPEG-curcumin solutions (2.5 mg/kg). Organs of interest (kidney, spleen, heart, lungs, liver, skin, and pancreas) were collected, rinsed with PBS, and weighed after 3 h of drug administration. Fluorescence imaging for Ce6-curcumin derivatives was determined using the FOBI imaging system (Neo-Science, Suwon, South Korea) for determining their ex vivo organ distributions. Then, the tissues were mixed with lysis buffer, homogenized by using a tissue homogenizer and centrifuged at 9000 rpm for 15 min to extract Ce6 and Ce6-curcumin derivatives. The supernatant was collected, and the Ce6 and Ce6-curcumin-conjugate content was analyzed using a Spark Multimode Microplate Reader (Tecan Trading AG, Männedorf, Switzerland) spectrophotometrically at 660 nm. The fluorescence intensity was normalized to the tissue weight.

### 4.12. Statistical Analysis

Obtained data were expressed as mean ± standard deviation (SD) and analyzed by one-way analysis of variance (ANOVA) followed by Tukey’s post hoc test with the aid of GraphPad Prism software (version 5.01, Inc., 2007, San Diego, CA, USA). *p* values less than 0.05 were considered statistically significant.

## 5. Conclusions

In summary, we have developed Ce6-curcumin derivatives to determine their anti-aging effect with PDT in Hs68 and BALB/c 3T3 cells. PDT with Ce6-diPEG-curcumin, among the other derivatives, showed lesser cytotoxicity, higher collagen production, decreased MMPs, and proinflammatory molecule expression via inhibition of the MAPK/AP-1/NF-κB signaling pathway. Interestingly, we also found that Ce6-diPEG-curcumin and other derivatives showed strong antioxidant effects in fibroblasts, which might be due to the synergistic effect of curcumin with Ce6-mediated PDT. Similarly, propane and diPEG derivatives were bound to the collagenase with the lowest energies of −7.2 and −7.3 kcal/mol, respectively. Therefore, Ce6-diPEG-curcumin derivative-PDT could be a potential therapy for their ameliorative effects against UVB-induced photoaging. However, additional research, particularly in animal models, is required to comprehend and unravel the molecular mechanisms underlying the antiphotoaging effects of Ce6-diPEG-curcumin derivatives-PDT. 

## Figures and Tables

**Table 1:** IC50 values of Ce6 and Ce6-curcumin derivatives in dark and LED conditions showing cell viability of Hs68 cells.

Compounds	Dark Toxicity (IC50, nM)	Phototoxicity (IC50, nM)
Ce6	≥1000	615
Ce6-propane-curcumin	≥1000	≥1000
Ce6-hexane-curcumin	≥1000	≥1000
Ce6-diPEG-curcumin	≥1000	692.6

**Table 2:** EC50 of Ce6 and Ce6-curcumin derivatives under dark and LED conditions for ABTS radical scavenging ability.

Compounds	Dark Condition (EC50, μM)	LED Condition (EC50, μM)
Ce6	13.58 ± 4.14	19.81 ± 1.57
Ce6-propane-curcumin	18.90 ± 3.04	34.92 ± 8.16
Ce6-hexane-curcumin	22.36 ± 4.66	36.34 ± 4.02
Ce6-diPEG-curcumin	15.51 ± 0.01	22.33 ± 2.62
5-ALA	60.28 ± 9.51	-
Curcumin	9.62 ± 0.005	-

Each scale value is shown as mean (n = 3) ± SD and was analyzed by one-way ANOVA with Tukey’s post hoc test. *p* < 0.01, compared with the ABTS scavenging radical scavenging ability of Ce6.

**Table 3:** Antioxidant activity by ORAC assay.

Compounds	Dark Condition (TE, μM)		LED Condition (TE, μM)	
	1	10	1	10
Ce6	18.5	47.72	22.11	53.85
Ce6-propane-curcumin	20.11	26.83	2.78	30.66
Ce6-hexane-curcumin	15.97	20.19	2.26	24.17
Ce6-diPEG-curcumin	20.85	30.32	0.85	27.98
5-ALA	ND	ND	-	-
Curcumin	38.56	56.54	-	-

Note: TE = Trolox equivalent; ND = Not detected. Each scale value is shown as the mean ± SD of a single experiment.

## Data Availability

All the data are contained within the article and supplementary material.

## Supplementary Materials

The supporting information can be downloaded at: https://www.mdpi.com/article/10.3390/ijms241713468/s1.
