# Supplementary material for: Chlorin E6-Curcumin-Mediated Photodynamic Therapy Promotes an Anti-Photoaging Effect in UVB-Irradiated Fibroblasts
Source: Int J Mol Sci. 2023 Aug 30;24(17):13468. doi: 10.3390/ijms241713468 (PMC10487708; doi:10.3390/ijms241713468)
Supplement: Supplementary file 1 [file ijms-24-13468-s001.zip › ijms-2543925-supplementary.pdf]

Chlorin e6-curcumin-mediated photodynamic therapy promotes an anti-photoaging effect in UVB-irradiated fibroblasts

Til Bahadur Thapa Magar[1], Shyam Kumar Mallik[1], Pallavi Gurung, Junmo Lim, Young-Tak Kim, Rajeev Shrestha, and Yong-Wan Kim*

Dongsung Cancer Center, Dongsung Biopharmaceutical, Daegu 41061, Republic of Korea

*Corresponding author: thomas06@hanmail.net

[1]Authors are equal contributors

Table of contents:

1. **Biodistribution of Ce6 in different time intervals**
2. **Pharmacokinetics of Ce6**
3. **Evaluation study of 3T3 NRU assay.**
4. **Molecular docking of Ce6-curcumin derivatives to MMP-2**

1. Biodistribution of Ce6 at different time intervals

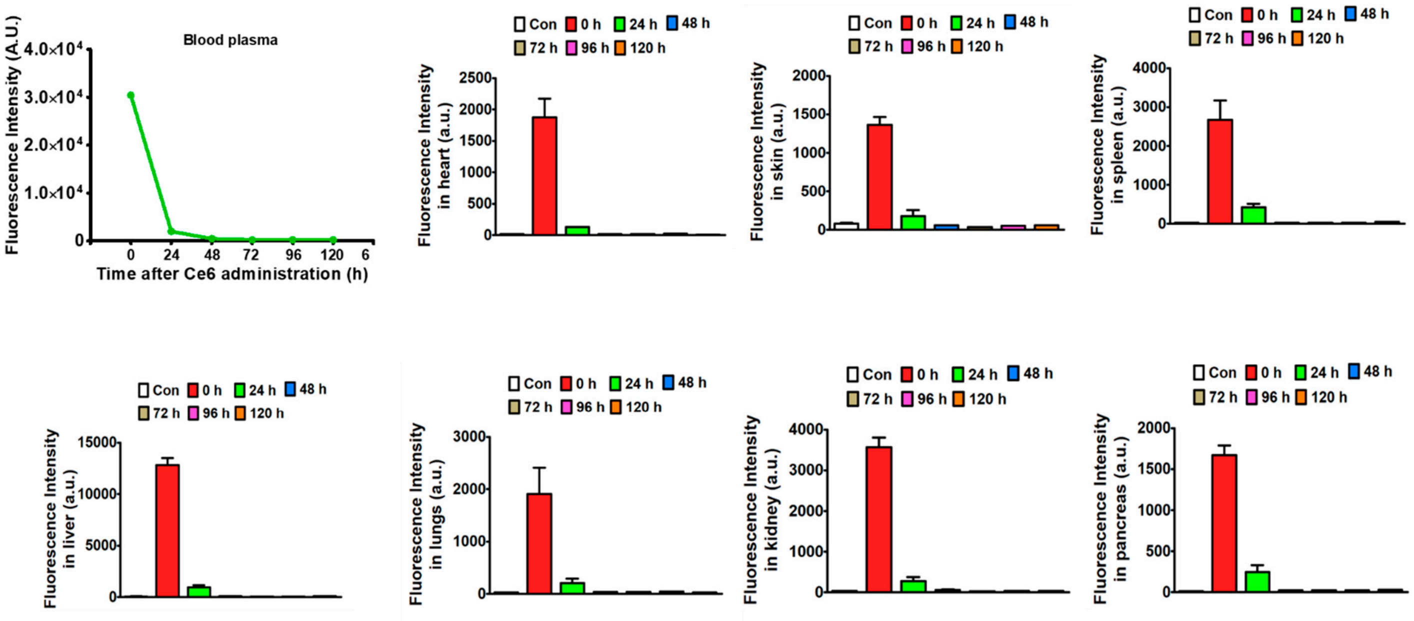

Figure S1. Time-dependent fluorescence intensity changes in plasma, heart, skin, spleen, liver, lungs, kidney, and pancreas following intravenous injection of Ce6 (2.5 mg/kg).

2. Pharmacokinetics of Ce6

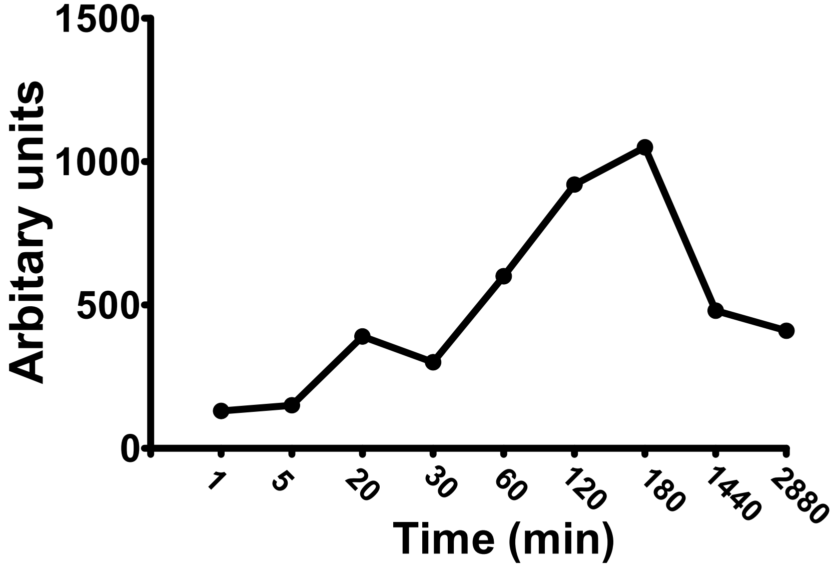

Figure S2. Changes in fluorescence intensity with time (min) in tumor tissue of rat after iv injection of Ce6 at the doses of 1 mg/kg (n=3).

3. Evaluation study of 3T3 NRU assay

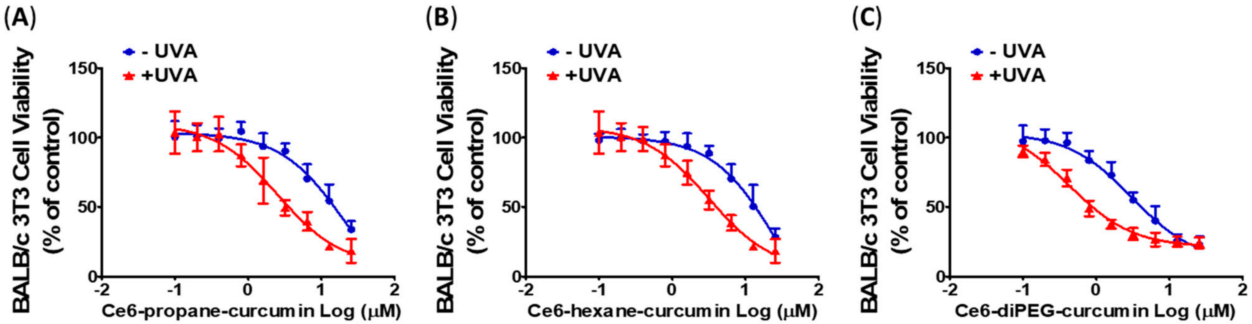

Figure S3: Effects of Ce6-curcumin conjugate treatments on BALB/c 3T3 cell under UVA- and UVA+ conditions and were detected by the Neutral Red method (3T3 NRU). Phototoxicity profiles of A) Ce6-propane-curcumin, B) Ce6-hexane-curcumin, and C) Ce6-diPEG-curcumin conjugates in BALB/c 3T3 cells exposed to a dose of 5 J/cm^2 of UVA (UVA+) while the other plate was incubated in dark (UVA-). After 24 h, cell viability was obtained by Neutral Red Uptake for 3 h. Data are the representation of the mean ± SD of three independent experiments.

Table S1. IC50 values of Ce6-curcumin-derivatives in UVA- and UVA+ conditions showing cell viability of BALB/c 3T3 fibroblast cells.

**Compounds**	**BALB/c 3T3 (IC50, µM)**			
	**UVA-**	**UVA+**	**PIF**	**PPH**
Ce6-propane-curcumin	15.20	3.56	4.26	NP
Ce6-hexane-curcumin	13.59	4.13	3.29	NP
Ce6-diPEG-curcumin	4.13	0.89	4.64	NP

Note: PIF = Photo-irritancy factor (UVA-/UVA+), PPH = Predicted phototoxic hazard based on PIF, and NP = Non-phototoxic (PIF < 5)

4. Molecular docking of Ce6-curcumin derivatives to MMP-2

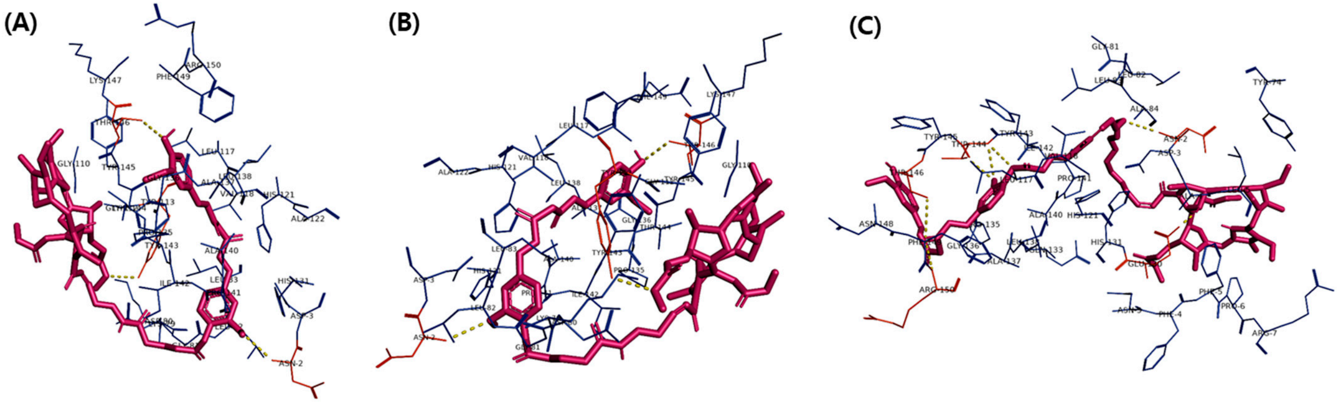

Ce6-Propane-Curcumin
LBE: -9.4 kcal/mol
Pocket volume: 677Å

Ce6-Hexane-Curcumin
LBE: -9.4 kcal/mol
Pocket volume: 677Å

Ce6-diPEG-Curcumin
LBE: -8.4 kcal/mol
Pocket volume: 677Å

Figure S4. Binding affinity of (A) Ce6-propane-curcumin, (B) Ce6-hexane-curcumin, and (C) Ce6-diPEG-curcumin conjugates to the MMP-2 (PDB code: 7XGJ). Lowest binding energy (LBE) and pocket volume were specified. Amino acids (AA) with polar and non-polar interaction were marked red and blue color respectively (hydrogen bonds were represented as yellow dotted lines).
